# Effect of Vaccination on Pneumococci Isolated from the Nasopharynx of Healthy Children and the Middle Ear of Children with Otitis Media in Iceland

**DOI:** 10.1128/JCM.01046-18

**Published:** 2018-11-27

**Authors:** Sigríður J. Quirk, Gunnsteinn Haraldsson, Helga Erlendsdóttir, Martha Á. Hjálmarsdóttir, Andries J. van Tonder, Birgir Hrafnkelsson, Samuel Sigurdsson, Stephen D. Bentley, Ásgeir Haraldsson, Angela B. Brueggemann, Karl G. Kristinsson

**Affiliations:** a University of Iceland, Faculty of Medicine, Reykjavík, Iceland; b Landspitali University Hospital, Department of Clinical Microbiology, Reykjavík, Iceland; c BioMedical Centre of the University of Iceland, Reykjavik, Iceland; d Parasites and Microbes, Wellcome Sanger Institute, Hinxton, United Kingdom; e University of Iceland, Department of Mathematics, Reykjavik, Iceland; f Children's Hospital Iceland, Reykjavík, Iceland; g Nuffield Department of Medicine, University of Oxford, Oxford, United Kingdom; h Department of Medicine, Imperial College London, London, United Kingdom; University of Iowa College of Medicine

**Keywords:** Iceland, *Streptococcus pneumoniae*, carriage, epidemiology, molecular epidemiology, otitis media, pneumococcus, vaccination, vaccine

## Abstract

Vaccination with pneumococcal conjugate vaccines (PCVs) disrupts the pneumococcal population. Our aim was to determine the impact of the 10-valent PCV on the serotypes, genetic lineages, and antimicrobial susceptibility of pneumococci isolated from children in Iceland. Pneumococci were collected between 2009 and 2017 from the nasopharynges of healthy children attending 15 day care centers and from the middle ears (MEs) of children with acute otitis media from the greater Reykjavik capital area. Isolates were serotyped and tested for antimicrobial susceptibility. Whole-genome sequencing (WGS) was performed on alternate isolates from 2009 to 2014, and serotypes and multilocus sequence types (STs) were extracted from the WGS data. Two study periods were defined: 2009 to 2011 (PreVac) and 2012 to 2017 (PostVac). The overall nasopharyngeal carriage rate was similar between the two periods (67.3% PreVac and 61.5% PostVac, *P* = 0.090). Vaccine-type (VT) pneumococci decreased and nonvaccine-type (NVT) pneumococci (serotypes 6C, 15A, 15B/C, 21, 22F, 23A, 23B, 35F, and 35B) significantly increased in different age strata post-PCV introduction. The total number of pneumococci recovered from ME samples significantly decreased as did the proportion that were VTs, although NVT pneumococci (6C, 15B/C, 23A, and 23B) increased significantly. Most serotype 6C pneumococci were multidrug resistant (MDR). Serotype 19F was the predominant serotype associated with MEs, and it significantly decreased post-PCV introduction: these isolates were predominantly MDR and of the Taiwan^19F^-14 PMEN lineage. Overall, the nasopharyngeal carriage rate remained constant and the number of ME-associated pneumococci decreased significantly post-PCV introduction; however, there was a concomitant and statistically significant shift from VTs to NVTs in both collections of pneumococci.

## Abstract (precis)

Vaccination with pneumococcal conjugate vaccines (PCVs) disrupts the pneumococcal population. Our aim was to determine the impact of the 10-valent PCV on the serotypes, genetic lineages, and antimicrobial susceptibility of pneumococci isolated from children in Iceland.

## INTRODUCTION

*Streptococcus pneumoniae* is an important human pathogen that can cause relatively mild upper respiratory tract infections, such as acute otitis media (AOM), or more severe infections, such as pneumonia and invasive pneumococcal disease (IPD) (1). Pneumococci frequently colonize the nasopharynges of humans, especially children attending day care centers (DCCs) (2, 3), and asymptomatic carriage occurs at least once prior to the age of 2 years (4). Nasopharyngeal colonization precedes pneumococcal disease, is a major factor for horizontal transmission within the community, especially among young children (5), and reflects the pneumococcal strains circulating in the community (6). Pneumococci are among the most frequent causes of bacterial AOM (7–9), which is the most common bacterial infection in children under 3 years of age (10). Pneumococcal conjugate vaccines (PCVs) interrupt the transmission of antibiotic-resistant pneumococci and thus decrease the burden of disease caused by antibiotic-resistant isolates in immunized children. Therefore, studies of the impact of these vaccines on antibiotic resistance and serotype distribution should focus both on pneumococci from nasopharyngeal carriage and from middle ears of children of similar ages with AOM (11).

Current PCVs target only a limited number of serotypes, especially those commonly causing IPD in young children (12–14). PCVs have been implemented in the infant vaccine immunization program in over 100 countries (15), which has resulted in a decrease of IPD caused by vaccine serotypes (VTs) in vaccinated children and other age groups due to herd immunity (16–18). Among healthy children, PCV implementation has also resulted in serotype replacement in carriage where VTs have been replaced with nonvaccine serotypes, meaning that the total carriage rate has remained constant (19–21). Pneumococcal carriage has been monitored and recorded in Iceland for the past 2 decades (22–24). The carriage rates have been consistent over time, ranging from 50% to 70% in healthy preschool children 1 to <7 years of age (25). The 10-valent PCV (PHiD-CV [Synflorix; GSK]), which directly targets 10 serotypes, was introduced into the national pediatric immunization program in Iceland in April 2011 in a 2-plus-1 schedule without catch-up. No other pneumococcal vaccine was previously included. The aim of this study was to assess the impact of PHiD-CV on the distribution of pneumococcal serotypes and genetic lineages among pneumococci from the nasopharynges of healthy children and the middle ears of children with otitis media and assess any changes in antimicrobial resistance rates after vaccine implementation.

## MATERIALS AND METHODS

### DCC study and bacterial isolates.

Nasopharyngeal swabs were taken from healthy children 1 to <7 years of age in March every year from 2009 to 2017 after informed consent was obtained from the parents. The children were attending 15 DCCs, chosen to be representative of the greater Reykjavík area. The period 2009 to 2011 was defined as the prevaccination period (PreVac), and the years 2012 to 2017 were the postvaccine implementation period (PostVac). The number of nasopharyngeal swabs taken from each age group is listed in Table S1 in the supplemental material. The samples were selectively cultured for pneumococci on blood agar containing 5 μg/ml gentamicin and incubated anaerobically (26). Very few children attended public DCCs before the age of 12 months, and no children <1 year of age were sampled in this study.

### Middle ear study and bacterial isolates.

All pneumococci isolated from middle ear (ME) samples from children 0 to <7 years of age with otitis media submitted to the Department of Clinical Microbiology, Landspitali University Hospital, between 1 January 2009 and 30 September 2017 were included in this study. This is the primary microbiology laboratory for the greater Reykjavík capital area. It provides services for individuals from the rural area and visiting specialists in the capital, and it is the reference laboratory for the whole country (approximately 85% of the population for ME samples).

The primary service area for the Landspitali University Hospital was considered to be within 100 km driving distance from the hospital, and the population demographic information for this referral region was obtained from Statistics Iceland (www.statice.is). The average population sizes for children 0 to <7 years of age in the referral region during the study period were 23,747 children PreVac and 24,083 children PostVac (approximately 66% of all children in Iceland 0 to <7 years of age). A detailed listing of the populations according to age groups can be seen in Table S2. When two or more pneumococcal isolates of the same phenotype were identified from the same patient within 30 days, only one isolate was included in the analyses.

### Serotyping.

Serotypes were determined for all available isolates with the Immulex pool antisera (State Serum Institute, Copenhagen, Denmark) and/or by multiplex PCR (mPCR), according to previously published methods (27–30). The mPCR scheme included 78 sets of serogroup/serotype-specific primer pairs and two primer pairs for a positive internal control: *cpsA* for the capsular locus and *lytA* for autolysin. Serotypes of serogroup 6 were identified using previously described PCR methods (31–33). Nontypeable (nonencapsulated S. pneumoniae [NESp]) isolates, i.e., those that were negative for *cpsA* and positive for *lytA*, were tested for the *cpsB* gene, which is essential for capsulation (34), according to a previously published PCR method (35). For those pneumococcal genomes that were sequenced, seqSerotyper (https://github.com/avantonder/seqSerotyper) was used to extract the serotypes from sequence data (36).

### DNA extraction and whole-genome sequencing.

Every other pneumococcal isolate from nasopharyngeal and ME samples from the period 2009 to 2014 was selected for whole-genome sequencing (WGS). DNA was extracted using the Promega Maxwell 16 platform, and the DNA extracts were sequenced on the Illumina HiSeq 2000. WGS data were assembled using Velvet (37) before SSPACE and GapFiller were used to improve the assemblies and close gaps (38, 39). The final assembled genomes were uploaded into a Bacterial Isolate Genome Sequence Database (BIGSdb) along with associated metadata (40). The multilocus sequence type (ST) of each isolate was extracted from the WGS data using BIGSdb and the PubMLST database (http://pubmlst.org/spneumoniae/). STs were assigned to clonal complexes (CCs) via Phyloviz (41).

### Genomic analyses.

Prokka was used to predict the coding sequences in each genome (42). The resulting annotation files in gff format were then used as the input for Roary and clustered using a sequence identity threshold of 90% (43). The core genome was estimated using a Bayesian core genome model and a threshold of 99.9% for nasopharyngeal isolates and 99.8% for ME isolates (44). The core genes in both sample groups were extracted and aligned using MAFFT (45). FastTree was used to construct phylogenetic trees, and ClonalFrameML (46) was used to reconstruct the trees to account for recombination. The final phylogenetic trees were then annotated using iTOL (47).

### Antibiotic susceptibility testing.

All isolates were tested for antimicrobial susceptibility to chloramphenicol, erythromycin, tetracycline, trimethoprim-sulfamethoxazole, and clindamycin by disk diffusion tests. Oxacillin disks (1 μg) were used to screen for penicillin resistance; isolates susceptible to oxacillin were considered to be susceptible to penicillin. For oxacillin-resistant isolates, the MICs to penicillin and ceftriaxone (ME isolates only) were measured using the Etest (bioMérieux, France) (48). Penicillin-nonsusceptible pneumococci (PNSP) were defined as isolates having a MIC of >0.06 mg/liter, while resistant pneumococci were isolates having a MIC of >2 mg/liter. Multidrug resistance (MDR) was defined as nonsusceptibility to three or more classes of antimicrobials (regardless of penicillin nonsusceptibility). The susceptibility testing was performed according to the methods and criteria of the European Committee on Antimicrobial Susceptibility Testing (EUCAST) (49).

### Statistical analyses.

A likelihood ratio test (50) was used to test the null hypothesis of equal rates when comparing the rate (r_1_) of a certain serotype, CC, or ST in a given age group PreVac to the rate (r_2_) of the same serotype, CC, or ST in the same age group PostVac. The test is based on the assumption that the counts of the serotype, CC, or ST in the two periods are independent and both follow Poisson distributions. It is assumed that the mean of the counts PreVac is equal to the rate r_1_ times the total number of individuals in the age group PreVac. The mean of the counts PostVac is equal to the rate r_2_ times the total number of individuals in the age group PostVac. The asymptotic distribution of the likelihood ratio test statistics is a chi-square distribution with one degree of freedom under the null hypothesis. Microsoft Excel was used to calculate the test statistics and the corresponding *P* values.

The two-sided Fisher's exact test was used to calculate the *P* values for antimicrobial resistance by using the statistical software R, version 3.3.2. The level of significance for all tests was ≤0.05. The Simpson diversity index was used to calculate the diversity of STs (51).

### Ethics.

The study was approved by The National Bioethics Committee (VSNb2013010015/03.07) and the appropriate authorities at the Landspitali University Hospital and the day care centers.

## RESULTS

### Nasopharyngeal samples from children attending DCCs.

A total of 4,461 nasopharyngeal swabs were collected (450 to 550 samples each year): 1,380 PreVac and 3,081 PostVac (Table 1). The median age of the children who were sampled was 4.1 years. The cultures yielded 3,029 pneumococcal isolates, and 250 children carried two pneumococcal strains. Nine isolates were excluded (not viable/not stored), yielding 3,020 isolates: 991 (32.8%) PreVac and 2,029 (67.2%) PostVac. The carriage rates were 67.3% PreVac and 61.5% PostVac (*P* = 0.090). Overall, 51.8% (*n* = 1,563) of the isolates were collected from children 4 to <7 years of age, and the fewest isolates, 3.5% (*n* = 103), were from the children 1 to <2 years of age (see Table S3 in the supplemental material). The genomes of 987 (49.2%) pneumococcal isolates from 2009 to 2014 were sequenced.

**TABLE 1:** Serotype distribution each study year in nasopharyngeal samples among children 1 to <7 years old PreVac (2009 to 2011) and PostVac (2012 to 2017)

Serotype or sample type	No. of isolates										2009 to 2011 (*n*/1,000 samples)	No. of isolates PostVac	2012 to 2017 (*n*/1,000 samples)	*P* value
	2009	2010	2011	2012	2013	2014	2015	2016	2017	PreVac				
3	18	26	24	11	13	20	32	25	12	68	49.3	113	36.7	0.052
4	2	0	0	0	0	0	0	0	0	2	1.4	0	0	0.096
6A	38	24	25	56	23	16	21	23	11	87	63.0	150	48.7	0.051
6B	50	45	14	17	16	26	9	1	2	109	79.0	71	23.0	<0.001
6C	3	4	2	0	11	7	31	49	53	9	6.5	151	49.0	<0.001
9V	12	3	3	1	0	0	0	0	0	18	13.0	1	0.3	<0.001
9A	0	0	1	0	1	0	0	0	0	1	0.7	1	0.3	0.619
9N	6	0	3	3	4	6	5	1	0	9	6.5	19	6.2	0.873
10	0	0	0	0	0	0	3	5	0	0	0	8	2.6	0.052
10A	5	0	0	2	1	8	3	1	8	5	3.6	23	7.5	0.131
10B	0	0	0	0	0	7	0	0	0	0	0	7	2.3	0.075
11A	18	19	21	22	10	38	37	24	5	58	42.0	136	44.1	0.756
13	0	0	0	0	1	0	0	0	0	0	0	1	0.3	0.691
14	26	36	12	8	9	9	3	0	0	74	53.6	29	9.4	<0.001
15	0	0	1	0	0	0	1	1	1	1	0.7	4	1.3	0.385
15A	0	0	0	0	0	1	14	11	9	0	0	35	11.4	<0.001
15B/C	21	6	10	29	27	29	24	22	15	37	26.8	146	47.4	<0.001
16F	3	8	13	3	2	4	5	5	6	24	17.4	25	8.1	0.008
17	0	0	0	0	0	0	1	0	0	0	0	1	0.3	0.691
18C	0	0	0	0	0	0	2	0	0	0	0	2	0.6	0.477
19	15	12	4	7	6	3	3	0	0	31	22.5	19	6.2	<0.001
19F	30	28	29	22	28	21	13	3	0	87	63.0	87	28.2	<0.001
19A	47	16	26	12	26	29	22	31	25	89	64.5	145	47.1	0.020
21	2	1	2	4	15	17	14	23	13	5	3.6	86	27.9	<0.001
22F	3	2	2	3	17	31	4	9	5	7	5.1	69	22.4	<0.001
23	0	0	0	0	0	0	1	2	1	0	0	4	1.3	0.227
23F	50	47	28	39	30	7	17	1	3	125	90.6	97	31.5	<0.001
23A	12	10	7	6	22	24	16	35	6	29	21.0	109	35.4	0.009
23B	1	0	0	9	15	25	41	34	28	1	0.7	152	49.3	<0.001
24F	0	0	0	0	0	0	0	0	4	0	0	4	1.3	0.227
29	3	0	0	2	0	0	0	0	0	3	2.2	2	0.6	0.210
31	2	0	0	0	0	0	0	1	5	2	1.4	6	1.9	0.765
33	2	2	1	4	2	4	0	1	0	5	3.6	11	3.6	0.954
33F	3	2	5	1	1	2	4	8	1	10	7.2	17	5.5	0.492
33_Hybrid	0	0	0	0	0	4	0	0	0	0	0	4	1.3	0.227
35F	0	0	0	3	15	18	16	4	0	0	0	56	18.2	<0.001
35B	1	1	4	0	17	12	13	2	13	6	4.3	57	18.5	<0.001
38	0	9	10	2	5	3	3	1	3	19	12.8	17	5.5	0.007
Other serotypes^*a*^	0	1	1	0	1	1	11	17	19	2	1.4	49	15.9	<0.001
NESp^*b*^	21	22	25	18	16	22	22	14	24	68	49.3	116	37.7	0.075
Total	394	324	273	284	334	394	391	354	272	991	718.1	2,029	658.6	<0.001
VT^*c*^	185	171	90	94	89	66	45	5	5	446	323.2	304	98.7	<0.001
NVT^*d*^	209	153	183	190	245	328	346	349	267	545	394.9	1,725	559.9	<0.001
All NP^*e*^ samples	516	444	420	465	471	566	533	540	506	1380		3,081		
Samples of positive Pn^*f*^ (%)	76.4	73.0	65.0	61.1	70.9	69.6	73.4	65.6	53.8	71.8		65.9		

a Serotypes other than those included in the multiplex PCR panel of the study.

b NESp, nonencapsulated S. pneumoniae.

c Serotypes detected in the study that are included in PHiD-CV (4, 6B, 9V, 14, 18C, 19F, and 23F).

d Serotypes that are not included in PHiD-CV.

e NP, nasopharyngeal samples.

f Pn, pneumococci.

### Serotypes.

A total of 36 different serotypes were detected: 27 PreVac and 35 PostVac. Overall, the numbers of isolates of serotypes included in PHiD-CV (vaccine type [VT]) decreased between the two periods (*P* < 0.001) (Table 1). VT pneumococci were most common in 2010 (171/324 [52.8%]) and least common in 2016 (5/354 [1.4%]) (Table 1). The numbers of isolates of serotypes not included in PHiD-CV (nonvaccine type [NVT]) increased from PreVac (*n* = 545, 395.2/1,000 samples) to PostVac (*n* = 1,725, 559.9/1,000 samples; *P* < 0.001) (Table 1). NVT pneumococci were most common in 2016 (349/354 [98.6%]) and least common in 2010 (153/324 [47.2%]) (Table 1).

In children 1 to <2 years of age, only one NVT, serotype 23A, increased from PreVac (*n* = 0, 0/1,000 samples) to PostVac (*n* = 7, 95.9/1,000 samples; *P* = 0.005). The NVTs that increased PostVac and were the most prevalent in children 2 to <4 years of age were of serotypes 6C (*n* = 82, 65.0/1,000 samples; *P* < 0.001), 15B/C (*n* = 81, 64.2/1,000 samples; *P* < 0.001), and 23B (*n* = 69, 54.7/1,000 samples; *P* < 0.001) (Table S3).

The NVTs that increased PostVac in children 4 to <7 years of age were serotypes 23B (*n* = 80, 45.8/1,000 samples; *P* < 0.001), 6C (*n* = 56, 32.1/1,000 samples; *P* < 0.001), and 21 (*n* = 53, 30.4/1,000 samples; *P* < 0.001). Serotype 23B was only detected PostVac among children 4 to <7 years of age (Table S3).

### MLST/CC.

**FIG 1:**
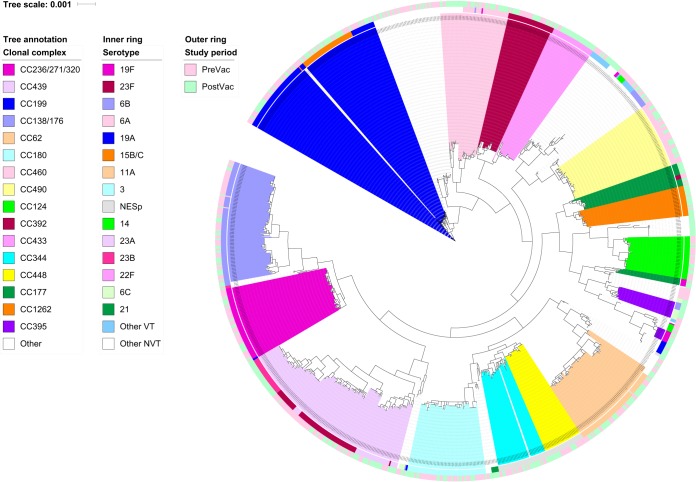
Phylogenetic tree created from 1,066 full-length coding loci found in 99.9% of 987 genomes from carriage samples and annotated with CC designations. Serotypes (inner circle) and study periods (outer circle) are also presented.

Among the 987 sequenced isolates, 47 CCs (35 CCs PreVac and 41 CCs PostVac) and 104 STs (66 STs PreVac and 83 STs PostVac) were detected, and 12 CCs and 43 STs were unique to nasopharyngeal isolates. The Simpson diversity index of the STs was 0.97 for both periods. A phylogenetic tree was created with the concatenated sequences of 1,066 full-length coding loci found in 99.9% of the nasopharyngeal carriage pneumococcal genomes. The tree was annotated with CC designations and serotypes (Fig. 1).

CC439^23F/A/B^ was the most common CC in both study periods. Initially, 77.6% of the isolates of CC439^23F/A/B^ were VT serotype 23F, but PostVac, 42.0% were NVT serotype 23B; however, serotype 23B was rare prior to vaccine introduction (Fig. 1 and Table S4). Between the two periods, the prevalence of CC433^22F^ increased from two isolates (1.5/1,000 samples) to 30 (20.0/1,000 samples; *P* < 0.001) (Fig. 1 and Table S4), and similarly, CC1262^15B/C^ increased from three isolates (2.2/1,000 samples) to 18 (12.0/1,000 samples; *P* = 0.032) (Fig. 1 and Table S4).

### Antimicrobial resistance.

The overall prevalence of PNSP among nasopharyngeal isolates did not change significantly between the two study periods: 15.0% (*n* = 149/991) versus 16.7% (338/2,029; *P* = 0.268) (Table 2). Erythromycin-resistant pneumococci decreased from 17.6% to 13.7% (*P* = 0.007). The overall prevalence of MDR pneumococci decreased slightly but significantly between the periods, from 15.2% (*n* = 151/991) to 12.4% (*n* = 251/2,029; *P* = 0.030) (Table 2). Before PHiD-CV was introduced, 85.2% of PNSP pneumococci were also MDR, and this was reduced to 70.1% (*P* < 0.001). The most prevalent PNSP and MDR isolates were of serotype 19F PreVac and NESp PostVac (Table 2).

**TABLE 2:** Most common PNSP and MDR serotypes in nasopharyngeal samples PreVac (2009 to 2011) and PostVac (2012 to 2017)

Serotype or sample type	PNSP					MDR Pn^*a*^				
	PreVac		PostVac		*P* value	PreVac		PostVac		*P* value
	*n*	%	*n*	%		*n*	%	*n*	%	
19F	74	49.7	59	17.5	<0.001	73	48.3	60	23.9	<0.001
NESp^*b*^	34	22.8	85	25.1	0.657	35	23.2	85	33.9	0.025
6B	12	8.1	4	1.2	<0.001	16	10.6	5	2.0	<0.001
14	11	7.4	1	0.3	<0.001	3	2.0	1	0.4	0.087
19A	6	4.0	35	10.4	0.021	3	2.0	9	3.6	0.516
6C	3	2.0	46	13.6	<0.001	4	2.6	47	18.7	<0.001
15A	0	0	29	8.6	<0.001	0	0	29	11.6	<0.001
23B	0	0	32	9.5	<0.001	0	0	1	0.4	1.000
35B	0	0	25	7.1	<0.001	0	0	0	0	NC^*c*^
Other PNSP/MDR^*d*^	9	6.0	22	6.5	1.000	4	2.6	6	2.4	1.000
Total	149		338		0.269	151		251		0.030

a Pn, pneumococci.

b NESp, nonencapsulated S. pneumoniae.

c NC, not calculated.

d Other less prevalent serotypes of PNSP and MDR pneumococci.

Among the PNSP serotype 19F isolates, 92.5% belonged to CC236/271/320^19F^ (Fig. 1 and Table S4). Serotype 19A PNSP increased from 4.0% to 10.4% of isolates (*P* = 0.021) (Table 2), and the isolates were predominantly members of CC199^19A,15B/C^ (Fig. 1 and Table S4). PNSP with serotypes 6C (also MDR) and 35B were also detected. Serotype 6C belonged to CC315^6B/C^. Serotype 35B PNSP were only detected after vaccine introduction, and they were members of CC198^35B^, which was not previously detected in carriage (Table 2, Fig. 1, and Table S4). The proportion of MDR NESp isolates increased between the two study periods from 23.2% to 33.9% (*P* = 0.025) (Table 2), and 82.4% of MDR NESp PostVac isolates were members of CC344^NT^ (Fig. 1 and Table S4).

### Middle ear samples from AOM.

The Department of Clinical Microbiology received 6,651 ME samples during the study period. The total annual number of ME samples decreased from 966 samples in 2009 to 421 by the end of September 2017 (Table 3). Among all 6,651 samples, 994 were positive for pneumococci and 18 isolates were excluded, as they were not stored or not viable, leaving a total of 976 isolates for further analysis. The annual number of pneumococcal isolates from ME decreased from 197 (8.5/1,000 children aged 0 to <7 years) in 2009 to 44 (1.9/1,000 children) in 2016, and by the end of September 2017, the number of isolates was 23 (1.0/1,000 children) (Table 3). The median age of the children from which the ME isolates were obtained was 1.5 years, and 69.1% (*n* = 674) of the isolates were collected from the youngest age group (0 to <2 years of age). The average annual number of isolates in the youngest age group (0 to <2 years) decreased from 133.7 (18.1/1,000 children aged 0 to <2 years per year) PreVac to 45.5 (6.9/1,000 children aged 0 to <2 years per year) PostVac (*P* = 0.020) (see Table S5). The genomes of 441 (50.6%) pneumococcal isolates from 2009 to 2014 were sequenced.

**TABLE 3:** Serotype distribution each study year in ME samples among children 0 to <7 years old PreVac (2009 to 2011) and PostVac (2012 to 2017)

Serotype or sample type	No. of isolates										2009 to 2011 (avg/yr)	No. of isolates PostVac	2012 to 2017 (avg/yr)	*P* value
	2009	2010	2011	2012	2013	2014	2015	2016	2017^*a*^	PreVac				
3	3	2	7	3	9	4	0	0	0	12	4.0	16	2.7	0.635
4	1	0	0	0	0	0	0	0	0	1	0.3	0	0	NC^*b*^
6A	14	22	11	13	12	2	1	1	0	47	15.7	29	4.8	0.159
6B	17	14	6	8	2	1	0	0	0	37	12.3	11	1.8	0.010
6C	0	0	1	2	10	10	7	8	4	1	0.3	41	6.8	<0.001
9N	0	0	1	0	0	0	0	0	0	1	0.3	0	0	NC
9V	4	2	1	0	0	0	0	0	0	7	2.3	0	0	NC
10B	0	0	0	0	1	0	0	0	0	0	0	1	0.2	NC
11A	1	4	3	6	3	4	1	2	1	8	2.7	17	2.8	0.239
14	14	13	10	1	4	1	0	0	0	37	12.3	6	1.0	<0.001
15A	0	0	0	0	1	1	0	1	3	0	0	6	1.0	NC
15B/C	2	2	1	15	22	2	5	8	1	5	1.7	53	8.8	<0.001
16F	1	1	0	0	0	0	0	1	0	2	0.7	1	0.2	0.695
17	0	1	0	0	0	0	0	0	0	1	0.3	0	0	NC
18C	1	1	0	1	0	0	0	0	0	2	0.7	1	0.2	0.695
19F	85	69	88	33	27	5	0	2	0	242	80.7	67	11.2	<0.001
19A	17	13	5	8	6	5	1	1	1	35	11.7	22	3.7	0.240
19C	1	0	0	0	0	0	0	0	0	1	0.3	0	0	NC
21	0	0	0	3	5	1	1	2	1	0	0	13	2.2	NC
22F	0	0	0	1	1	2	0	0	0	0	0	4	0.7	NC
23F	29	21	23	9	6	3	0	0	0	73	24.3	18	3.0	<0.001
23A	1	1	0	2	5	1	8	6	1	2	0.7	23	3.8	0.003
23B	0	0	1	5	3	2	1	2	2	1	0.3	15	2.5	0.012
24F	0	0	1	0	0	0	0	2	0	1	0.3	2	0.3	0.707
33F	0	0	4	3	4	3	0	1	0	4	1.3	11	1.8	0.231
35F	0	1	0	0	3	0	1	0	0	1	0.3	4	0.7	0.365
35B	0	0	0	3	2	2	1	1	0	0	0	9	1.5	NC
38	1	0	1	0	0	0	0	0	0	2	0.7	0	0	NC
Other serotypes^*c*^	4	10	5	13	12	11	11	6	9	19	6.3	62	10.3	0.001
NESp^*d*^	1	0	0	1	0	0	0	0	0	1	0.3	1	0.2	0.995
Total	197	177	169	130	138	60	38	44	23	543	181	433	72.2	0.014
VT^*e*^	151	120	128	52	39	10	0	2	0	399	133.0	96	16.0	<0.001
NVT^*f*^	46	57	41	78	99	50	38	42	23	144	49.0	337	56.2	<0.001
All ME samples	966	926	951	849	894	687	505	452	421	2,843	947.7	3,808	634.7	
Samples positive for Pn^*g*^ (%)	20.4	19.1	17.8	15.3	15.4	8.7	7.5	9.7	5.5	19.1		11.4		

a From 1 January to 30 September 2017.

b NC, not calculated.

c Serotypes other than those included in the multiplex PCR panel of the study.

d NESp, nonencapsulated S. pneumoniae.

e Serotypes detected in the study that are included in PHiD-CV (4, 6B, 9V, 14, 18C, 19F, and 23F).

f Serotypes detected in the study that are not included in PHiD-CV.

g Pn, pneumococci.

### Serotypes.

Overall, 894/976 (91.6%) pneumococcal isolates from ME samples were successfully serotyped, but 82 isolates (8.4%) were of serotypes other than those included in the mPCR scheme. Twenty-eight serotypes were detected overall: 23 PreVac and 22 PostVac. The numbers of VT pneumococci decreased significantly between the two periods (*P* < 0.001) (Table 3). The numbers of VT pneumococci decreased significantly in the two younger age groups (children 0 to <2 years, *P* < 0.001; and 2 to <4 years of age, *P* = 0.005), while there was no change between the periods for the oldest age group (children 4 to <7 years of age; *P* = 0.450) (Table S5).

The NVT pneumococci that increased PostVac in children aged 0 to <2 years were serotypes 15B/C (*n* = 1 to *n* = 40; *P* < 0.001), 6C (*n* = 1 to *n* = 29; *P* < 0.001), 23A (*n* = 2 to *n* = 18; *P* = 0.007), and 23B (*n* = 1 to *n* = 10; *P* = 0.042). Serotype 15B/C was the only NVT that increased in children 2 to <4 years of age (*n* = 1 to *n* = 11; *P* = 0.042), and serotype 6C (*n* = 12) was only detected PostVac within that age group (Table S5).

### MLST/CC.

**FIG 2:**
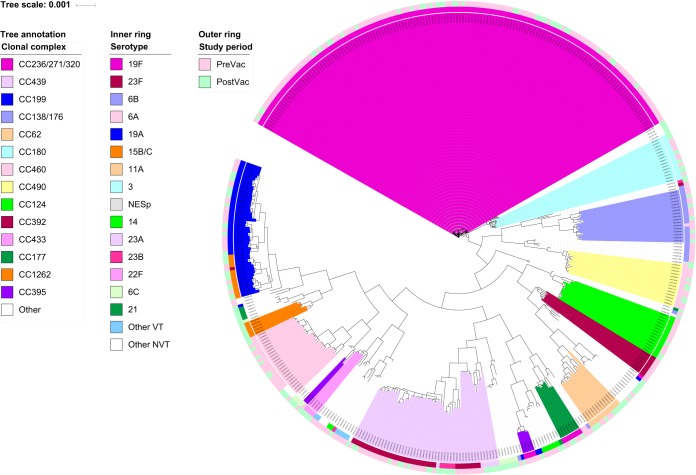
Phylogenetic tree created from 1,250 full-length coding loci found in 99.8% of the 441 genomes from ME samples and annotated with CC designations. Serotypes (inner circle) and the study periods (outer circle) are also presented.

Among the 441 sequenced isolates, 41 CCs (29 CCs PreVac and 31 CCs PostVac) and 86 STs (55 STs PreVac and 52 STs PostVac) were detected, and 7 CCs and 24 STs were unique to ME isolates. The STs of two isolates could not be determined as a full-length allele was missing from the WGS data, but these isolates were members of CC180 and CC236/271/320. The Simpson diversity indices of the STs were 0.91 PreVac and 0.96 PostVac. A phylogenetic tree was created with the concatenated sequences of 1,250 full-length coding loci found in 99.8% of the ME pneumococcal genomes. The tree was annotated with CC designations and serotypes (Fig. 2).

Fewer VT CCs were detected PostVac (6 CCs) than PreVac (18 CCs), and fewer STs were detected within the CCs. Six STs of VT isolates were detected within CC439^23F^ PreVac, compared to only one ST PostVac (Fig. 2 and Table S6). CC236/271/320^19F^ was the most common CC in both study periods: 41.8% (115/275, 4.8/1,000 children) PreVac, but it decreased in prevalence to 22.3% (37/166, 1.5/1,000 children) PostVac (*P* < 0.001) (Fig. 2 and Table S6). Between the periods, CC315^6B/C^ increased from one serotype 6B isolate to six serotype 6C isolates (*P* = 0.05). CC1262^15B/C^, CC193^21^, and CC1816^35B^, which contained NVTs, were only detected PostVac in ME samples, but CC1262^15B/C^ was detected in low numbers in nasopharyngeal carriage PreVac. One isolate of NVT serotype 23B PostVac belonged to CC156/162^9V^, a typically VT lineage (Fig. 2 and Table S6).

### Antimicrobial resistance.

The overall prevalence of PNSP among ME isolates decreased between the two study periods (48.1% versus 28.4%; *P* < 0.001). The resistance to erythromycin decreased between the two periods (49.7% versus 29.8% of isolates; *P* < 0.001), and the prevalence of MDR pneumococci decreased also (49.2% versus 29.8%; *P* < 0.001). Before the introduction of PHiD-CV, 98.1% (56/261) of PNSP were also MDR, and this was reduced to 91.9% (113/123; *P* < 0.001). PNSP and MDR isolates were mainly serotype 19F in both study periods (Table 4). Among the PNSP serotype 19F isolates, 89.2% belonged to CC236/271/320^19F^ (Fig. 2 and Table S6). NVT serotype 6C amounted to 15.4% of PNSP (*P* < 0.001) and 23.3% of MDR pneumococci (*P* < 0.001) detected PostVac (Table 4). The serotype 6C PNSP/MDR isolates were not detected before vaccine implementation and were members of CC315^6B,6C^.

**TABLE 4:** Most common PNSP and MDR serotypes in ME samples PreVac (2009 to 2011) and PostVac (2012 to 2017)

Serotype	PNSP					MDR Pn^*a*^				
	PreVac		PostVac		*P* value	PreVac		PostVac		*P* value
	*n*	%	*n*	%		*n*	%	*n*	%	
19F	231	88.6	66	53.7	<0.001	232	86.9	65	50.4	<0.001
6B	9	3.4	3	2.4	0.759	16	6.0	3	2.3	0.017
14	6	2.3	2	1.6	1.000	5	1.9	2	1.6	0.472
19A	4	1.5	9	7.3	0.006	2	0.7	9	7.0	0.014
23F	4	1.5	0	0	0.311	4	1.5	0	0	0.134
6C	0	0	19	15.4	<0.001	0	0	30	23.3	<0.001
15A	0	0	6	4.9	0.001	0	0	6	4.7	0.007
23B	1	0.4	5	4.1	0.014	1	0.4	1	0.8	1.000
Other PNSP/MDR Pn^*b*^	6	2.3	13	10.6	0.001	7	2.6	13	10.1	0.040
Total	261		123		<0.001	267		129		<0.001

a Pn, pneumococci.

b Other less prevalent serotypes of PNSP and MDR pneumococci.

Serotype 19A PNSP increased from 1.5% to 7.3% (*P* = 0.006), and MDR serotype 19A increased from 0.7% to 7.0% (*P* = 0.014) (Table 4). Serotype 19A PNSP/MDR pneumococci were members of various CCs (Fig. 2 and Table S6). NVT PNSP with serotypes 15A and 23B also increased after vaccine introduction, and they were members of CC63^15A^ and CC338^23B^, respectively. NVT serotype 15A PNSP (also MDR) was only detected after vaccination in ME isolates (Table 4, Fig. 2, and Table S6).

### Comparison of serotypes in nasopharyngeal isolates from carriage and ME isolates from children with AOM 1 to <4 years of age.

Overall, 2,291 pneumococcal isolates were obtained from children 1 to <4 years of age: 1,457 isolates from nasopharyngeal samples from carriage (*n* = 493 PreVac and *n* = 964 PostVac) and 834 isolates from AOM ME samples (*n* = 467 PreVac and *n* = 367 PostVac).

**FIG 3:**
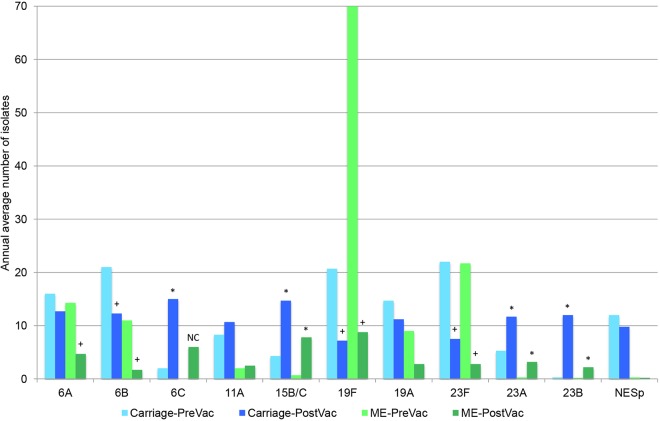
The annual average numbers of the most prevalent serotypes and NESp isolates detected in carriage versus ME samples among children 1 <4 years of age PreVac (2009 to 2011) and PostVac (2012 to 2017). +, decreased significantly between the two study periods; *, increased significantly between the two study periods; Nc, not calculated; NESp, nonencapsulated S. pneumoniae.

The same serotypes were among the most prevalent serotypes PreVac in both sample groups, and the levels of serotype replacement PostVac were similar in both groups (Table 5, Fig. 3). One isolate of VT serotype 23F was detected in carriage among children 1 to <4 years of age in 2017, and no VTs were detected in children with AOM within the same age group after 2016. Serotype 6A decreased slightly PostVac among children with AOM but not in carriage (*P* = 0.050) (Fig. 3). Serotypes 6C, 15B/C, 23A, and 23B increased (Fig. 3).

**TABLE 5:** The most common VTs and NVTs in carriage and ME samples from children 1 to <4 years old PreVac (2009 to 2011) and PostVac (2012 to 2017)

Serotype or sample type	PreVac					PostVac				
	Carriage		ME		*P* value	Carriage		ME		*P* value
	No. of isolates	% positive cultures	No. of isolates	% positive cultures		No. of isolates	% positive cultures	No. of isolates	% positive cultures	
6A	48	9.7	43	9.2	0.766	76	7.9	28	7.6	0.889
6B	63	12.8	33	7.1	0.003	37	3.8	10	2.7	0.332
6C	6	1.2	0	0	0.018	90	9.3	36	9.8	0.696
11A	25	5.1	6	1.3	<0.001	64	6.6	15	4.1	0.074
15B/C	13	2.6	2	0.4	0.005	88	9.1	47	12.8	0.051
19F	63	12.8	215	46.0	<0.001	43	4.5	53	14.4	<0.001
19A	44	8.9	27	5.8	0.061	67	7.0	17	4.6	0.117
23F	67	13.6	65	13.9	0.830	45	4.7	17	4.6	0.993
23A	16	3.2	1	0.2	<0.001	70	7.3	19	5.2	0.172
23B	1	0.2	1	0.2	0.975	72	7.5	13	3.5	0.006
Less prevalent serotypes	111	22.5	73	15.6	NC^*a*^	255	26.5	111	30.2	NC
NESp^*b*^	36	7.3	1	0.2	<0.001	59	6.1	1	0.3	<0.001
Total	493	100	467	100	NC	964	100	367	100	NC
VT^*c*^	256	51.9	353	75.6	<0.001	141	14.6	87	23.7	<0.001
NVT^*d*^	237	48.1	114	24.4	<0.001	823	85.4	280	76.3	<0.001

a NC, not calculated.

b NESp, nonencapsulated S. pneumoniae.

c Serotypes detected in the study that are included in PHiD-CV (4, 6B, 9V, 14, 18C, 19F, and 23F).

d Serotypes detected in the study that are not included in PHiD-CV.

VT serotype 19F was more frequently found in AOM than in carriage in children 1 to <4 years of age in both study periods: 32.1% (268/834) of ME isolates were serotype 19F compared to 7.2% (105/1,457) of carriage isolates (*P* < 0.001). The NVT serotype 23B was more frequently found in carriage than in AOM in children 1 to <4 years of age: 5.0% (73/1,457) of carriage isolates were serotype 23B compared to only 1.7% (14/834) of the ME isolates (*P* = 0.006). Serotype 6C was prevalent in both carriage and AOM among children 1 to <4 years of age, but MDR was more common in ME isolates (*P* < 0.001). NESp isolates were more frequently found in carriage than among children with AOM in both study periods (*P* < 0.001) (Table 5).

## DISCUSSION

This study shows a significant reduction of VTs in nasopharyngeal carriage of healthy children and in ME samples in children <7 years old 6 years after PHiD-CV implementation in Iceland. The total number of pneumococci isolated from the nasopharynges of children remained unchanged after vaccination due to serotype replacement by NVTs; however, at the same time, the total number of ME isolates decreased significantly. The ME samples in our study were most often from children with ruptured tympanic membranes as a consequence of AOM or from children with tympanic tubes and discharge from the middle ear as a consequence of inflammation in the middle ear. Consequently, we postulate that the decrease in the number of samples from the ME most likely reflects a decrease in the burden of AOM (52). Certain serotypes are associated with nasopharyngeal carriage in healthy children, while others are more prone to cause disease (53, 54); however, the NVTs that have replaced the VTs following routine vaccination may, to a great extent, express a low invasive disease potential (55).

It was confirmed in our study that the distribution of serotypes and genetic lineages in nasopharyngeal carriage in children reflected those identified from the discharges from ears of children with AOM (56). Furthermore, the diversity of the pneumococcal STs did not change between the periods.

The same serotypes and genetic lineages were often found in nasopharyngeal and ME samples in children 1 to <4 years of age, both PreVac and PostVac but often in different proportions. There was a slight but significant decrease in serotype 6A among children with AOM, which has been described in IPD among all ages following PHiD-CV vaccination (57). Serotypes 6C, 23A, and 23B were not affected by the vaccination, as these serotypes increased significantly in both sample groups, which has also been documented elsewhere (21, 58–62).

Serotype 19F was particularly “otogenic,” as it was four times more prevalent in ME samples than in nasopharyngeal samples. The main serotype 19F in CC236/270/320^19F^ harbors genes for pili (both PI-1 and PI-2), which likely contributed to the adherence to the mucosa of the middle ear (63). Serotype 6B had a preference for nasopharyngeal colonization PreVac, while the same was true for serotype 23B PostVac. This finding is in concordance with other reports where serotype 23B was associated with persistent carriage and posed a lower risk for IPD (55, 64).

The NESp isolates were largely associated with carriage in both study periods but extremely rare in AOM. However, while the prevalence of NESp did not change significantly after vaccination in carriage, MDR among NESp isolates increased significantly.

In our study, only one VT isolate was detected between 2014 and 2016 in ME samples among children 1 to <4 years of age, whereas 11 VT isolates were detected between 2014 and 2017 in the nasopharynges of healthy children of the same ages. This indicates that PHiD-CV may be more effective in preventing AOM (measured as fewer positive ME samples) than in preventing nasopharyngeal carriage. This is important, as AOM caused by pneumococci is more severe than that caused by other common pathogens (65, 66).

The increase of the MDR serotype 6C isolates PostVac in both nasopharynx and ME samples is of concern. Other researchers have also described an increase in serotype 6C following the introduction of PCV7, especially in non-IPD among children (67). We rarely detected isolates of serotype 6C PreVac; however, in a recent study, serotype 6C was only detected among vaccinated children with non-IPD after PHiD-CV implementation (68). Serotype 6C isolates detected PostVac most often belonged to CC315^6B^ and ST386^6C^ (a double locus variant [DLV] of PMEN Poland^6B^-20). The MDR ST386^6C^ was detected in Spain 6 years after PCV7 implementation (69), and other countries have also reported the emergence of this lineage in nasopharyngeal carriage and IPD following the implementation of PHiD-CV (62). This lineage might be derived from a capsular switch from serotype 6B to serotype 6C, as has previously been reported by our group (36).

The majority of serotype 23B isolates belonged to CC439/ST439^23B^ (single locus variant [SLV] of PMEN Tennessee^23F^-4); however, this lineage was only detected PostVac in both sample groups in our study. CC439/ST439^23B^ was present, although uncommon, in Germany prior to the implementation of vaccinations but increased after vaccine implementation. This lineage has also been documented in other countries worldwide (70).

One ME isolate of serotype 23B belonged to CC156/162 and ST162 (an SLV of PMEN Spain^9V^-3). This was the only isolate of this lineage detected PostVac in ME isolates. Serotype switch variants of the related ST156 expressing serotypes 9V, 9A, 14, 19F, and 11A have also been reported (71). Pneumococci have the ability to change their capsular serotype by exchanging the capsular locus genes (72). The expansion of preexisting lineages and their variants, such as CC315^6B/C^ and CC439^23F/B^, may be more likely following PCV vaccination than the emergence of new lineages (70).

Iceland offers a unique opportunity for researching vaccine effects for several reasons. The reference laboratory at the Department of Clinical Microbiology, Landspitali University Hospital, serves approximately 85% of the country for pneumococcus-positive samples and stores all isolates (at −80°C). Furthermore, carriage studies have been conducted within the same DCCs using the same methodology throughout the study period. PCVs were not part of the routine infant immunization program before PHiD-CV implementation in 2011. Since then, vaccine acceptance and the uptake of PCVs have been high (73, 74). In our study, we analyzed a large number of pneumococcal isolates representative of the Icelandic population. Furthermore, one third of the pneumococcal isolates were subjected to WGS, which gives a good overview of the composition of genetic lineages in the country. However, fluctuations in serotypes and genotypes are known among pneumococci, even without the selective pressure of PCVs (75, 76).

In conclusion, PHiD-CV implementation eliminated VTs in the MEs of children with otitis media within 5 years. The carriage rate of pneumococci in healthy children remained constant between the periods due to serotype replacement of NVTs, but the total number of ME isolates decreased significantly PostVac. Serotype 23B and NESp had a preference for nasopharyngeal carriage among children 1 to <4 years of age. Multidrug resistance among serotype 6C was more common in ME samples among children 1 to <4 years of age than in nasopharyngeal carriage samples PostVac.

## Supplementary Material

Supplemental file 1

Supplemental file 2

Supplemental file 3

Supplemental file 4

Supplemental file 5

Supplemental file 6
