# Supplementary material for: Effect of Vaccination on Pneumococci Isolated from the Nasopharynx of Healthy Children and the Middle Ear of Children with Otitis Media in Iceland
Source: J Clin Microbiol. 2018 Nov 27;56(12):e01046-18. doi: 10.1128/JCM.01046-18 (PMC6258863; doi:10.1128/JCM.01046-18)
Supplement: Supplemental file 1 [file zjm012186168s1.pdf]

Table S1. Number of nasopharyngeal swabs from children attending DCCs according to each age group, PreVac (2009-2011) and PostVac (2012-2017).

Age group	PreVac	PostVac
1 to <2 years	81	73
2 to <4 years	567	1,262
4 to <7 years	732	1,746
Total 1 to <7 years	1,380	3,081
