# Supplementary material for: Effect of Vaccination on Pneumococci Isolated from the Nasopharynx of Healthy Children and the Middle Ear of Children with Otitis Media in Iceland
Source: J Clin Microbiol. 2018 Nov 27;56(12):e01046-18. doi: 10.1128/JCM.01046-18 (PMC6258863; doi:10.1128/JCM.01046-18)
Supplement: Supplemental file 2 [file zjm012186168s2.pdf]

Table S2. Number of children in the uptake area within each age group for ME samples PreVac (2009-2011) and PostVac (2012-2017).

Age group	PreVac	PostVac
0 to <2 years	7,385	6,621
2 to <4 years	6,818	6,973
4 to <7 years	9,544	10,490
Total 0 to <7 years	23,747	24,083
