# Supplementary material for: Effect of Vaccination on Pneumococci Isolated from the Nasopharynx of Healthy Children and the Middle Ear of Children with Otitis Media in Iceland
Source: J Clin Microbiol. 2018 Nov 27;56(12):e01046-18. doi: 10.1128/JCM.01046-18 (PMC6258863; doi:10.1128/JCM.01046-18)
Supplement: Supplemental file 3 [file zjm012186168s3.pdf]

Table S3. Serotype distribution within each age group PreVac (2009-2011) and PostVac (2012-2017) in nasopharyngeal samples.

	**1 to <2 years**			**2 to <4 years**			**4 to <7 years**		
**Serotype**	**PreVac; 2009-11 /1000 samples[a] (n)**	**PostVac; 2012-17 /1000 samples (n)**	**PreVac vs. PostVac p-value**	**PreVac; 2009-11 /1000 samples (n)**	**PostVac; 2012-17 /1000 samples (n)**	**PreVac vs. PostVac p-value**	**PreVac; 2009-11 /1000 samples (n)**	**PostVac; 2012-17 /1000 samples (n)**	**PreVac vs. PostVac p-value**
3	24.7 (2)	0 (0)	0.275	17.6 (10)	19.0 (24)	0.859	76.5 (56)	51.0 (89)	0.016
4	0 (0)	0 (0)	NC[b]	0 (0)	0 (0)	NC	2.7 (2)	0 (0)	0.359
6A	61.7 (5)	41.1 (3)	0.594	75.8 (43)	57.8 (73)	0.149	53.3 (39)	42.4 (74)	0.240
6B	148.1 (12)	0 (0)	<0.001	89.9 (51)	29.3 (37)	<0.001	62.8 (46)	19.5 (34)	<0.001
6C	12.3 (1)	82.2 (6)	0.053	8.8 (5)	66.6 (84)	<0.001	4.1 (3)	34.9 (61)	<0.001
9V	12.3 (1)	0 (0)	0.526	5.3 (3)	0 (0)	0.029	19.1 (14)	0.6 (1)	<0.001
9A	0 (0)	0 (0)	NC	1.8 (1)	0.8 (1)	0.620	0 (0)	0 (0)	NC
9N	0 (0)	0 (0)	NC	3.5 (2)	4.0 (5)	0.932	9.6 (7)	8.0 (14)	0.691
10	0 (0)	0 (0)	NC	0 (0)	4.8 (6)	0.107	0 (0)	1.1 (2)	0.359
10A	0 (0)	0 (0)	NC	0 (0)	7.1 (9)	0.035	6.8 (5)	8.0 (14)	0.787
10B	0 (0)	0 (0)	NC	0 (0)	1.6 (2)	0.476	0 (0)	2.9 (5)	0.147
11A	0 (0)	54.8 (4)	0.043	44.1 (25)	47.5 (60)	0.757	45.1 (33)	41.2 (72)	0.659
13	0 (0)	0 (0)	NC	0 (0)	0 (0)	NC	0 (0)	0.6 (1)	0.517
14	37.0 (3)	0 (0)	0.143	89.9 (51)	10.3 (13)	<0.001	27.3 (20)	9.2 (16)	0.001
15	0 (0)	0 (0)	NC	1.8 (1)	2.4 (3)	0.664	0 (0)	0 (0)	NC
15A	0 (0)	13.7 (1)	0.291	0 (0)	10.3 (13)	0.020	0 (0)	12.0 (21)	0.036
15B/C	24.7 (2)	95.9 (7)	0.072	19.4 (11)	64.2 (81)	<0.001	32.8 (24)	33.2 (58)	0.969
16F	0 (0)	0 (0)	NC	14.1 (8)	9.5 (12)	0.391	21.9 (16)	7.4 (13)	0.004
17	0 (0)	0 (0)	NC	0 (0)	0 (0)	NC	0 (0)	0.6 (1)	0.517
18C	0 (0)	0 (0)	NC	8.8 (5)	2.4 (3)	0.079	35.5 (26)	9.2 (16)	<0.001
19	0 (0)	0 (0)	NC	0 (0)	0.8 (1)	0.690	0 (0)	0.6 (1)	0.517
19F	123.5 (10)	54.8 (4)	0.099	93.5 (53)	30.9 (39)	<0.001	32.8 (24)	25.2 (44)	0.296
19A	61.7 (5)	95.9 (7)	0.551	68.8 (39)	47.5 (60)	0.073	61.5 (45)	44.7 (78)	0.085
21	0 (0)	13.7 (1)	0.474	1.8 (1)	25.4 (32)	<0.001	5.5 (4)	30.4 (53)	<0.001
22F	0 (0)	0 (0)	NC	3.5 (2)	20.6 (26)	0.003	6.8 (5)	24.6 (43)	0.002
23	0 (0)	0 (0)	NC	0 (0)	1.6 (2)	0.476	1.4 (1)	0.6 (1)	0.528
23F	74.1 (6)	0 (0)	0.010	107.6 (61)	35.7 (45)	<0.001	79.2 (58)	29.8 (52)	<0.001
23A	0 (0)	95.9 (7)	0.004	28.2 (16)	49.9 (63)	0.037	17.8 (13)	22.3 (39)	0.480
23B	0 (0)	41.1 (3)	0.104	1.8 (1)	54.7 (69)	<0.001	0 (0)	45.8 (80)	<0.001
24F	0 (0)	0 (0)	NC	0 (0)	1.6 (2)	0.476	0 (0)	1.1 (2)	0.359
29	0 (0)	0 (0)	NC	1.8 (1)	0.8 (1)	0.062	2.7 (2)	1.1 (2)	0.420
31	0 (0)	0 (0)	NC	0 (0)	1.6 (2)	0.476	2.7 (2)	2.3 (4)	0.817
33	0 (0)	0 (0)	NC	7.1 (4)	6.3 (8)	0.841	1.4 (1)	2.3 (4)	0.711
33F	0 (0)	0 (0)	NC	8.8 (5)	1.6 (2)	0.037	6.8 (5)	8.6 (15)	0.787
33_Hybrid	0 (0)	0 (0)	NC	0 (0)	0.8 (1)	0.690	0 (0)	1.7 (3)	0.262
35F	0 (0)	0 (0)	NC	0 (0)	15.8 (20)	<0.001	0 (0)	20.6 (36)	<0.001
35B	0 (0)	54.8 (4)	0.043	5.3 (3)	19.8 (25)	0.022	4.1 (3)	16.0 (28)	0.016
38	0 (0)	0 (0)	NC	12.3 (7)	5.5 (7)	0.145	16.4 (12)	5.7 (10)	0.015
Other[c]	0 (0)	41.1 (3)	0.104	1.8 (1)	19.8 (25)	<0.001	1.4 (1)	12.6 (22)	<0.001
NESp[d]	37.0 (3)	54.8 (4)	0.623	58.2 (33)	43.6 (55)	0.182	43.7 (32)	32.6 (57)	0.183
**Total**	**617.3 (50)**	**726.0 (53)**	**0.270**	**781.3 (443)**	**721.9 (911)**	**0.011**	**680.3 (498)**	**610.0 (1065)**	**<0.001**
VT[e]	395.1 (32)	54.8 (4)	<0.001	395.1 (224)	108.6 (137)	<0.001	259.6 (190)	93.4 (163)	<0.001
NVT[f]	222.2 (18)	671.2 (49)	<0.001	386.2 (219)	613.3 (774)	<0.001	420.8 (308)	516.6 (902)	<0.001

[a]Per nasopharyngeal samples taken from each age group.

[b]NC: Not calculated.

[c]Other: Serotypes other than those included in the multiplex PCR panel of the study.
[d]NESp: Non-encapsulated *S pneumoniae*.
[e]VT: The serotypes detected in the study that are included in PHiD-CV (4, 6B, 9V, 14, 18C, 19F, 23F).
[f]NVT: Serotypes that are not included in PHiD-CV.
