# Supplementary material for: Effect of Vaccination on Pneumococci Isolated from the Nasopharynx of Healthy Children and the Middle Ear of Children with Otitis Media in Iceland
Source: J Clin Microbiol. 2018 Nov 27;56(12):e01046-18. doi: 10.1128/JCM.01046-18 (PMC6258863; doi:10.1128/JCM.01046-18)
Supplement: Supplemental file 4 [file zjm012186168s4.pdf]

Table S4. Serotypes, clonal complexes (CC) and multilocus sequence types (ST) detected PreVac (2009-2011) and PostVac (2012-2014) in nasopharyngeal samples from carriage. Arranged according to the most prevalent serotype/CC and STs detected PreVac.

Nasopharyngeal samples from carriage PreVac; 2009-11			Nasopharyngeal samples from carriage PostVac; 2012-14		
**Serotype (n)**	**CC (n)**	**ST (n: PMEN[a])**	**Serotype (n)**	**CC (n)**	**ST (n: PMEN)**
23F (64)	439 (45)	311 (18: DLV[b] Tennessee^{23F}-4), 36 (9)	23F (36)	439 (19)	311 (11: DLV Tennessee^{23F}-4), 36 (4)
		507 (9: DLV Tennessee^{23F}-4), 37 (6: Tennessee^{23F}-4)			42 (1: DLV Tennessee^{23F}-4)
		442 (1), 10353 (1) 10359 (1)			507 (1: DLV Tennessee^{23F}-4), 442 (1) 13126 (1)
	392 (16), 177 (3)	440 (16), 1877 (3: DLV Greece^{21}-30)		392 (16), 433 (1)	440 (16), 10368 (1)
6B (65)	138/176 (57), 90 (5)	176 (39: DLV Poland^{23F}-16), 138 (16), 90 (5: Spain^{6B}-2)	6B (40)	138/176[d] (26)	138 (16), 176[d] (10: DLV Poland^{23F}-16),
	315 (1)	315 (1: Poland^{6B}-20)		395 (5), 90 (4)	395 (5: SLV Portugal^{6A}-41), 90 (4: Spain^{6B}-2)
	396 (1), 460 (1)	1716 (1), 460 (1)		396 (3), 171 (2)	1716 (3), 639 (2)
19A (45)	199 (42)	667 (19: SLV[e] Netherlands^{15B}-37)	19A (37)	199 (27)	10360 (11: DLV Netherlands^{15B}-37)
		199 (11: Netherlands^{15B}-37)			199 (8: Netherlands^{15B}-37)
		10360 (12: DLV Netherlands^{15B}-37)			667 (8: SLV Netherlands^{15B}-37)
	3017 (2)	3017 (2)		Sing1801 (8), 346 (1)	1801 (8), 10365 (1)
	Sing1801[f] (1)	1801 (1)		236/271/320 (1)	320 (1: DLV Taiwan^{19F}-14)
19F (41)	236/271/320 (35)	3014 (22: DLV Taiwan^{19F}-14), 9165 (7: DLV Taiwan^{19F}-14)	19F (28)	236/271/320 (17)	3014 (10: DLV Taiwan^{19F}-14), 9165 (4: DLV Taiwan^{19F}-14)
		9458 (4: DLV Taiwan^{19F}-14), 271 (2: SLV Taiwan^{19F}-14)			9828 (2), 271 (1: SLV Taiwan^{19F}-14)
	156/162 (2), 15 (1)	162 (2: SLV Spain^{9V}-3), 3016 (1)		395 (7)	10358 (6), 425 (1)
	177 (1)	12986 (1: SLV Portugal^{19F}-21)			
	395 (1), 460 (1)	425 (1), 460 (1)		177 (4)	179 (3: SLV Portugal^{19F}-21), 51 (1: SLV Portugal^{19F}-21)
6A (41)	490 (24), 460 (9)	2221 (24), 460 (6), 65 (2), 2340 (1)	6A (45)	490 (22), 460 (21)	2221 (22), 460 (13), 65 (6), 2340 (2)
	15 (8)	3981 (5)		138/176 (2)	176 (2: DLV Poland^{23F}-16)
3 (34)	180 (34)	180 (27: Netherlands^{3}-31), 505 (7: DLV Netherlands^{3}-31)	3 (21)	180 (21)	180 (20: Netherlands^{3}-31), 505 (1: DLV Netherlands^{3}-31)
14 (30)	124 (21), 15 (4),	124 (21: Netherlands^{14}-35), 9 (4: England^{14}-9)	14 (10)	124 (9),	124 (8: Netherlands^{14}-35), 13127 (1: SLV Netherlands^{14}-35)
	143 (3), 156/162 (2)	143 (3), 2306 (2: SLV Spain^{9V}-3)		15 (1)	9 (1: England^{14}-9)
11A (24)	62 (24)	62 (24: DLV Netherlands^{8}-33)	11A (37)	62 (37)	62 (35: DLV Netherlands^{8}-33), 10345 (2)
15B/C (18)	199 (15), 1262 (3)	199 (15: Netherlands^{15B}-37), 1262 (3)	15B/C (44)	199 (26), 1262[c] (18)	199 (26: Netherlands^{15B}-37), 1262[c] (18)
16F (14)	30 (14)	30 (14)	16F (6)	30 (6)	2340 (2), 2042 (1)
38 (13)	393 (13)	393 (13)	38 (3)	393 (3)	393 (3)
23A (13)	439 (13)	42 (7: DLV Tennessee^{23F}-4), 436 (4: DLV Tennessee^{23F}-4)	23A (22)	439 (22)	42 (13: DLV Tennessee^{23F}-4), 190 (4: DLV Tennessee^{23F}-4)
		190 (1), 10349 (1: DLV Tennessee^{23F}-4)			438 (2), 2404 (2) 992 (1)
18C (10)	113 (8)	113 (5: Netherlands^{18C}-36), 116 (2: SLV Netherlands^{18C}-36)	18C (7)	113 (7)	113 (7: Netherlands^{18C}-36)
		110 (1: SLV Netherlands^{18C}-36)			
	102 (2)	1071 (2)			
9V (9)	156/162 (9)	162 (9: SLV Spain^{9V}-3)	9V (1)	156/162 (1)	162 (1: SLV Spain^{9V}-3)
33F (7)	100 (7)	100 (7)	33F (4)	100 (3), 717 (1)	100 (3), 717 (1)
9N (5)	66 (5)	66 (5: SLV Tennessee^{14}-18)	9N (5)	66 (5)	66 (5: SLV Tennessee^{14}-18)
6C (4)	315 (2), 395 (2)	386 (2: DLV Poland^{6B}-20), 1692 (2: DLV Portugal^{6A}-41)	6C (10)	395 (5)	1692 (4: DLV Portugal^{6A}-41), 1714 (1: DLV Portugal^{6A}-41)
				1379 (3), 315 (2)	1379 (3), 386 (2: DLV Poland^{6B}-20)
10A (3)	460 (2)	461 (2)	10A (2)	460 (2)	461 (1), 816 (1)
	5825/11845 (1)	11845 (1)			
21 (2)	177 (1)	1877 (2: DLV Greece^{21}-30)	21 (16)	177 (11), 432 (5)	1877 (11: DLV Greece^{21}-30), 432 (5)
22F (2)	433 (2)	433 (2)	22F (29)	433[c] (29)	433[c] (28), 13129 (1)
35B (2)	1816 (2)	1967 (2)	35B (16)	198 (11), 452 (2)	4346 (10), 198 (1), 452 (2)
				1816 (2)	1967 (2)
				Sing2690 (1)	2690 (1)
4 (1)	205 (1)	205 (1: Sweden^{4}-38)	4 (0)		
31 (1)	568 (1)	1601 (1)	31 (0)		
23B (1)	338 (1)	2372 (1)	23B (29)	439 (28), 338 (1)	439 (28: SLV Tennessee^{23F}-4), 1349 (1: DLV Colombia^{23F}-26)
10B (0)			10B (7)	Sing6524 (7)	6524 (7)

15A (0)			15A (1)	473 (1)	473 (1)
33_Hybrid (0)			33_Hybrid (4)	62 (4)	673 (4)
35F (0)			35F (12)	460 (12)	1635 (10), 446 (2)
NESp[g] (33)	344 (17)	10371 (12: SLV Norway^{NT}-42), 344 (4: Norway^{NT}-42)	NESp (33)	344 (17)	344 (8: Norway^{NT}-42), 10371 (6: SLV Norway^{NT}-42)
		4149 (1: SLV Norway^{NT}-42)			4145 (1: SLV Norway^{NT}-42)
					4149 (1: SLV Norway^{NT}-42), 13128 (1: SLV Norway^{NT}-42)
	448 (15), 62 (1)	448 (15: USA^{NT}-43), 62 (1: DLV Netherlands^{8}-33)		448 (13)	448 (12: USA^{NT}-43), 10373 (1: SLV USA^{NT}-43)
				Sing10375 (2)	10375 (2)
				Sing10346 (1)	10346 (1)

[a]PMEN: Pneumococcal molecular epidemiology network clone. [b]DLV: Double locus variant. [c]Significant changes from PreVac to PostVac ($p=0.001$). [d]Significant changes from PreVac to PostVac ($p<0.001$). [e]SLV: Single locus variant. [f]Sing: Singleton. [g]NESp: Non-encapsulated *S. pneumoniae*.
