# Supplementary material for: Effect of Vaccination on Pneumococci Isolated from the Nasopharynx of Healthy Children and the Middle Ear of Children with Otitis Media in Iceland
Source: J Clin Microbiol. 2018 Nov 27;56(12):e01046-18. doi: 10.1128/JCM.01046-18 (PMC6258863; doi:10.1128/JCM.01046-18)
Supplement: Supplemental file 5 [file zjm012186168s5.pdf]

Table S5. Serotype distribution within each age group PreVac (2009-2011) and PostVac (2012-2017) in ME samples.

Serotype	0 to < 2 years			2 to < 4 years			4 to < 7 years		
	PreVac; 2009-11	PostVac; 2012-17	PreVac vs. PostVac	PreVac; 2009-11	PostVac; 2012-17	PreVac vs. PostVac	PreVac; 2009-11	PostVac; 2012-17	PreVac vs. PostVac
	Average/year (n)	Average/year (n)	p-value	Average/year (n)	Average/year (n)	p-value	Average/year (n)	Average/year (n)	p-value
3	1.0 (3)	0.7 (4)	0.730	2.0 (6)	1.3 (8)	0.745	1.0 (3)	0.7 (4)	0.867
4	0 (0)	0 (0)	NC[a]	0 (0)	0 (0)	NC	0.3 (1)	0 (0)	NC
6A	10.7 (32)	2.5 (15)	0.159	5.0 (15)	2.2 (13)	0.773	0 (0)	0.2 (1)	NC
6B	8.3 (25)	0.5 (3)	0.004	3.7 (11)	1.0 (7)	0.512	0.3 (1)	0.2 (1)	0.965
6C	0.3 (1)	4.8 (29)	<0.001	0 (0)	2.0 (12)	NC	0 (0)	0 (0)	NC
9V	1.7 (5)	0 (0)	NC	0 (0)	0 (0)	NC	0 (0)	0 (0)	NC
9N	0.3 (1)	0 (0)	NC	0.7 (2)	0 (0)	NC	0 (0)	0 (0)	NC
10B	0 (0)	0 (0)	NC	0 (0)	0.2 (1)	NC	0 (0)	0 (0)	NC
11A	1.3 (4)	1.5 (9)	0.292	1.0 (3)	1.0 (7)	0.411	0.3 (1)	0.2 (1)	NC
14	9.0 (27)	0.5 (3)	0.004	3.0 (9)	0.5 (3)	0.233	0.3 (1)	0 (0)	NC
15A	0 (0)	0.7 (4)	NC	0 (0)	0.2 (1)	NC	0 (0)	0 (0)	NC
15B/C	0.3 (1)	6.7 (40)	<0.001	0.3 (1)	1.8 (11)	0.042	1.0 (3)	0.3 (2)	0.715
16F	0.3 (1)	0 (0)	NC	0.3 (1)	0 (0)	NC	0 (0)	0.2 (1)	NC
17	0.3 (1)	0 (0)	NC	0 (0)	0 (0)	NC	0 (0)	0 (0)	NC
18C	0.3 (1)	0.2 (1)	0.959	0 (0)	0 (0)	NC	0.3 (1)	0 (0)	NC
19F	67.7 (203)	7.8 (47)	<0.001	11.0 (33)	2.3 (14)	0.057	2.0 (6)	0.8 (5)	0.763
19A	8.7 (26)	2.3 (14)	0.302	3.0 (9)	1.0 (6)	0.589	0 (0)	0.3 (2)	NC
19C	0.3 (1)	0 (0)	NC	0 (0)	0 (0)	NC	0 (0)	0 (0)	NC
21	0 (0)	1.5 (9)	NC	0 (0)	0.5 (3)	NC	0 (0)	0.2 (1)	NC
22F	0 (0)	0.2 (1)	NC	0 (0)	0.3 (2)	NC	0 (0)	0.2 (1)	NC
23F	16.3 (49)	1.0 (6)	<0.001	7.7 (23)	1.8 (11)	0.157	0.3 (1)	0.2 (1)	NC
23A	0.7 (2)	3.0 (18)	0.007	0 (0)	0.5 (3)	NC	0 (0)	0.3 (2)	NC
23B	0.3 (1)	1.7 (10)	0.042	0 (0)	0.7 (4)	NC	0 (0)	0.2 (1)	NC
24F	0.3 (1)	0.2 (1)	NC	0 (0)	0.2 (1)	NC	0 (0)	0 (0)	NC
33F	1.0 (3)	1.7 (10)	0.150	0.3 (1)	0.2 (1)	0.992	0 (0)	0 (0)	NC
35F	0 (0)	0.3 (2)	NC	0.3 (1)	0.3 (2)	0.710	0 (0)	0 (0)	NC
35B	0 (0)	1.0 (6)	NC	0 (0)	0.5 (3)	NC	0 (0)	0 (0)	NC
38	0.7 (2)	0 (0)	NC	0 (0)	0 (0)	NC	0 (0)	0 (0)	NC
Other[b]	3.7 (11)	6.7 (40)	0.004	1.7 (5)	2.5 (15)	0.140	0.3 (1)	1.3 (8)	0.120
NESp[c]	0 (0)	0.2 (1)	NC	0.3 (1)	0 (0)	NC	0 (0)	0 (0)	NC
**Total**	**133.7 (401)**	**45.5 (273)**	**0.020**	**40.3 (121)**	**21.5 (129)**	**0.829**	**7.0 (21)**	**5.2 (31)**	**0.488**
VT[d]	103.3 (310)	10.0 (60)	<0.001	26.0 (78)	5.8 (35)	0.005	3.7 (11)	1.2 (7)	0.450
NVT[e]	30.3 (91)	35.5 (213)	<0.001	14.3 (43)	15.7 (94)	0.005	3.3 (10)	4.0 (24)	0.153

[a]NC: Not calculated.
[b]Other: Serotypes other than those included in the multiplex PCR panel of the study.
[c]NESp: Non-encapsulated *S. pneumoniae*.
[d]VT: Serotypes detected in the study that are included in PHiD-CV (4, 6B, 9V, 14, 18C, 19F, 23F).
[e]NVT: Serotypes detected in the study that are not included in PHiD-CV.
