# Supplementary material for: Effect of Vaccination on Pneumococci Isolated from the Nasopharynx of Healthy Children and the Middle Ear of Children with Otitis Media in Iceland
Source: J Clin Microbiol. 2018 Nov 27;56(12):e01046-18. doi: 10.1128/JCM.01046-18 (PMC6258863; doi:10.1128/JCM.01046-18)
Supplement: Supplemental file 6 [file zjm012186168s6.pdf]

**Table S6.** Serotypes, clonal complexes (CC) and multilocus sequence types (ST) detected PreVac (2009-2011) and PostVac (2012-2014) in ME samples. Arranged according to the most prevalent serotype/CC and STs detected PreVac.

| ME samples PreVac; 2009-2011 | | | ME samples PostVac; 2012-2014 | | |
|---|---|---|---|---|---|
| **Serotype (n)** | **CC (n)** | **ST (n: PMEN[a])** | **Serotype (n)** | **CC (n)** | **ST (n: PMEN)** |
| 19F (124) | 236/271/320 (115) | 3014 (76: DLV[b] Taiwan$^{19F}$-14) | 19F (40) | 236/271/320[c] (37) | 3014 (23: DLV Taiwan$^{19F}$-14) |
| | | 9165 (15: DLV Taiwan1$^{9F}$-14) | | | 9828 (10) |
| | | 9458 (13: SLV[d] Taiwan$^{19F}$-14) | | | 9165 (3: SLV Taiwan$^{19F}$-14) |
| | | 271 (4: SLV Taiwan$^{19F}$-14) | | | 9458 (1: DLV Taiwan$^{19F}$-14) |
| | | 1968 (2: DLV Taiwan$^{19F}$-14), 9828 (2) | | 177 (3) | 177 (1: Portugal$^{19F}$-21), |
| | | 10385 (2: DLV Taiwan$^{19F}$-14), unknown (1) | | | 179 (2: SLV Portugal$^{19F}$-21 |
| | 177 (4), 395 (4) | 179 (3: SLV Portugal$^{19F}$-21) | | | |
| | | 9827 (1: SLV Portugal$^{19F}$-21), 425 (4) | | | |
| | 63 (1) | 1545 (1: DLV Sweden$^{15A}$-25) | | | |
| 23F (37) | 439 (28) | 311 (16: DLV Tennessee$^{23F}$-4), 36 (6) | 23F (8) | 439 (8) | 311 (8: DLV Tennessee$^{23F}$-4) |
| | | 9829 (3), 37 (1: Tennessee$^{23F}$-4) | | | |
| | | 442 (1), 507 (1: DLV Tennessee$^{23F}$-4) | | | |
| | 392 (7), 199 (1) | 440 (7), 199 (1: Netherlands$^{15B}$-37) | | | |
| | 361 (1) | 277 (1) | | | |
| 6A (23) | 460 (12), 490 (8) | 460 (6), 65 (5), 2340 (1), 2221 (8) | 6A (13) | 460 (7), 490 (6) | 460 (5), 2340 (2), 2221 (5), 10029 (1) |
| | 15 (2), 176 (1) | 3981 (2), 176 (1: DLV-Poland$^{23F}$-16) | | 395 (1) | 395 (1: SLV Portugal$^{6A}$-41) |
| 19A (20) | 199 (19) | 667 (8: SLV-Netherlands$^{15B}$-37) | 19A (15) | 199 (11) | 667 (4: Netherlands$^{15B}$-37), 199 (4: Netherlands$^{15B}$-37), 10360 (3: DLV Netherlands$^{15B}$-37) |
| | | 199 (7: Netherlands$^{15B}$-37), 10360 (4: DLV-Netherlands$^{15B}$-37) | | 63 (1), 230 (1) | 63 (1: Sweden$^{15A}$-25), 2013 (1: DLV Denmark$^{14}$-32) |
| | Sing1801[e] (1) | 1801 (1) | | 346 (1) Sing1801 (1) | 10365 (1), 1801 (1) |
| 6B (21) | 138/176 (11) | 176 (6: DLV-Poland$^{23F}$-16), 138 (5) | 6B (5) | 138/176 (5) | 176 (2: Poland$^{23F}$-16), 10393 (1: DLV Poland$^{23F}$-16) |
| | 171 (1) 396 (1) | 639 (1), 1716 (1) | | | 138 (2) |
| | 90 (6), 135 (1) | 90 (6: Spain$^{6B}$-2), 135 (1) | | | |
| | 315 (1) | 315 (1: Poland$^{6B}$-20) | | | |
| 14 (18) | 124 (11) | 124 (11: Netherlands$^{14}$-35), | 14 (4) | 124[f] (3) | 124[f] (3: Netherlands$^{14}$-35) |
| | 15 (5) 156/162 | 9 (4: England$^{14}$-9), 1964 (1: DLV England$^{14}$-9), 2306 (2: SLV Spain$^{9V}$-3) | | 15 (1) | 1964 (1: DLV England$^{14}$-9) |
| 3 (12) | 180 (12) | 180 (12: Netherlands$^{3}$-31) | 3 (7) | 180 (7) | 180 (6: Netherlands$^{3}$-31), unknown (1) |
| 11A (4) | 62 (4) | 62 (4: DLV Netherlands$^{8}$-33) | 11A (8) | 62 (8) | 62 (7: DLV Netherlands$^{8}$-33), 1367 (1) |
| 9V (5) | 156/162 (5) | 162 (4: SLV: Spain$^{9V}$ -3), 1269 (1: DLV Spain$^{9V}$-3) | 9V (0) | | |
| 15B/C (3) | 199 (3) | 199 (2: Netherlands$^{15B}$-37), 2220 (1: SLV Netherlands$^{15B}$-37) | 15B/C (15) | 199 (10) | 199 (10: Netherlands$^{15B}$-37) |
| | | | | 1262 (5) | 1262 (4), 8711 (1) |
| 16F (2) | 30 (2) | 30 (2) | 16F (0) | | |
| 18C (2) | 113 (2) | 113 (1: Netherlands$^{18C}$-36), 116 (1: SLV Netherlands$^{18C}$-36) | 18C (0) | | |
| 33F (2) | 100 (1), 717 (1) | 100 (1), 717 (1) | 33F (5) | 100 (3), 717 (2) | 100 (3), 717 (2) |
| 38 (1) | 393 (1) | 393 (1) | 38 (0) | | |
| 23A (1) | 439 (1) | 42 (1: DLV Tennessee$^{23F}$-4) | 23A (6) | 439 (6) | 42 (3: DLV Tennessee$^{23F}$-4) |
| | | | | | 190 (1), 436 (1: DLV Tennessee$^{23F}$-4) |
| | | | | | 438 (1) |
| 23B (0) | | | 23B (7) | 439 (5), 156/162 (1) | 439 (5: SLV Tennessee$^{23F}$-4), 162 (1: SLV Spain$^{9V}$-3) |
| | | | | 338 (1) | 1349 (1: DLV Colombia$^{23F}$-26) |
| 24F (1) | 230 (1) | 230 (1: Denmark$^{14}$-32) | 24F (0) | | |
| 35F (1) | 460 (1) | 446 (1) | 35F (3) | 460 (3) | 1635 (3) |
| 6C (1) | 395 (1) | 1692 (1: DLV Portugal$^{6A}$-41) | 6C (10) | 315[g] (6) | 386 (5: DLV Poland$^{6B}$-20) |
| | | | | | 10362 (1) |
| | | | | 1379 (3), 177 (1) | 1379 (3), 1533 (1: SLV Greece$^{21}$-30) |
| 9N (1) | 66 (1) | 10344 (1: DLV Tennessee$^{14}$-18) | 9N (0) | | |
| 21 (0) | | | 21 (6) | 193 (4), 432 (1) | 1877 (4: DLV Greece$^{21}$-30), 432 (1) |

| | | | |
|---|---|---|---|
| | | Sing10356 (1) | 10356 (1) |
| 22F (0) | 22F (4) | 433 (4) | 433 (4) |
| 35B (0) | 35B (5) | 1816 (4), 198 (1) | 1967 (3), 10361 (1), 4346 (1) |
| 10B (0) | 10B (1) | Sing6524 (1) | 6524 (1) |
| 15A (0) | 15A (2) | 63 (2) | 63 (2: Sweden[15A]-25) |
| NESp[h] (0) | NESp (1) | 1182 (1) | 3691 (1) |
| | | | |

[a]PMEN: Pneumococcal molecular epidemiology network clone. [b]DLV: Double locus variant. [c]Significant changes from PreVac to PostVac (p<0.001). [d]SLV: Single locus variant. [e]Sing: Singleton. [f]Significant changes from PreVac to PostVac (p=0.024). [g]Significant changes from PreVac to PostVac (p=0.05). [h]NESp: Non-encapsulated *S. pneumoniae*.
